# Differentiation between Acute Skin Rejection in Allotransplantation and T-Cell Mediated Skin Inflammation Based on Gene Expression Analysis

**DOI:** 10.1155/2015/259160

**Published:** 2015-02-12

**Authors:** Dolores Wolfram, Evi M. Morandi, Nadine Eberhart, Theresa Hautz, Hubert Hackl, Bettina Zelger, Gregor Riede, Tanja Wachter, Sandrine Dubrac, Christian Ploner, Gerhard Pierer, Stefan Schneeberger

**Affiliations:** ^1^Department of Plastic, Reconstructive and Aesthetic Surgery, Medical University of Innsbruck, Anichstrasse 35, 6020 Innsbruck, Austria; ^2^Department of Visceral, Transplant and Thoracic Surgery, Medical University of Innsbruck, Anichstrasse 35, 6020 Innsbruck, Austria; ^3^Division of Bioinformatics, Biocenter, Medical University of Innsbruck, Innrain 80, 6020 Innsbruck, Austria; ^4^Department of Pathology, Medical University of Innsbruck, Muellerstrasse 44, 6020 Innsbruck, Austria; ^5^Department of Dermatology and Venereology, Medical University of Innsbruck, Anichstrasse 35, 6020 Innsbruck, Austria

## Abstract

Advances in microsurgical techniques and immunosuppressive medication have rendered transplantation of vascularized composite allografts possible, when autologous tissue is neither available nor sufficient for reconstruction. However, skin rejection and side effects of long-term immunosuppression still remain a major hurdle for wide adoption of this excellent reconstructive technique. Histopathologic changes during acute skin rejection in vascular composite allotransplantation often mimic inflammatory skin disorders and are hard to distinguish. Hence, the identification of diagnostic and therapeutic markers specific for skin rejection is of particular clinical need. Here we present novel markers allowing for early differentiation between rejection in hind limb allotransplantation and contact hypersensitivity. Assessment of Ccl7, Il18, and Il1b expression is most indicative of distinguishing skin rejection from skin inflammatory disorders. Gene expression levels varied significantly across skin types and regions, indicating localization specific mechanism of leukocyte migration and infiltration. Expression of Il12b, Il17a, and Il1b gene expression levels differed significantly between rejection and inflammation, independent of the skin type. In synopsis of the RNA expression profile and previously assessed protein expression, the Il1 family appears as a promising option for accurate skin rejection diagnosis and, as a following step, for development of novel rejection treatments.

## 1. Introduction

Vascularized composite allotransplantation (VCA) is a treatment option for patients suffering from limb loss or severe disfigurement, when conventional reconstructive options are neither available nor sufficient for reconstruction. While graft survival can be achieved by using modern immunosuppressive therapy, rejection of the skin remains a misunderstood hurdle for wider application of VCA.

Untreated rejection in VCA is characterized by an inflammatory cell mediated cytotoxic process, which progressively harms the epidermis and the junction between dermis and epidermis. Histological assessment of tissue biopsies represents the standard procedure for diagnosing skin rejection after VCA [1]. However it is difficult to make histology based differential diagnoses from inflammatory, infectious, and neoplastic dermatoses [2]. Another major drawback of the assessment of skin rejection by histology is the latency between initiation of rejection and diagnosis.

Similarities between acute skin rejection and T-cell mediated immune responses of the skin have been shown on molecular and cellular level [3, 4]. Hapten-induced contact hypersensitivity (CHS) and delayed type hypersensitivity (DTH) are considered as standard models for an antigen-specific T-lymphocyte mediated immune response [5, 6] mainly characterized by presence of CD4+ and CD8+ cells as well as elements of the innate immune system (e.g., natural killer cells). The infiltration of alloantigen-specific T-cells into the skin allograft has also been identified as a central element of acute skin rejection in VCA [7, 8]. In recent years, cytokines and in particular chemokines have emerged as potent stimulators of effector cell accumulation and activation during the elicitation phase of CHS and DTH reactions as well as during allograft rejection in solid organ transplantation [9, 10]. Our group has previously shown that the infiltration and migration of T-cells into the skin allograft are also orchestrated by a multitude of cytokines, chemokines, and adhesion molecules [7, 11].

We herein analyzed the gene expression profile of inflammatory mediators during vascularized composite allograft acute skin rejection and the elicitation phase of antigen-specific T-cell mediated skin inflammation in established rodent models. We aimed to compare the gene expression analyses between skin from rejected allografts and skin with contact hypersensitivity (CHS) and delayed type hypersensitivity (DTH) reactions. Finally, we wanted to clarify if localization specific mechanisms in hairy versus nonhairy skin influence leukocyte migration in skin rejection as well as in skin inflammation.

Understanding the regulation of cytokine networks in different skin types during rejection as well as the differentiation between acute rejection versus inflammatory skin diseases will help to understand the complex mechanisms of rejection, allow earlier diagnosis, and enable the development of new therapeutic strategies.

## 2. Methods

### 2.1. Animals

Eight- to ten-week-old (200–250 g) male brown Norway (BN) and Lewis rats (LEW) (Charles River Laboratories, Germany) were used as donors and recipients in the transplant setting. For the induction of hypersensitivity skin disorders, male LEW rats were applied. All animals received care in compliance with the “Principles of Laboratory Animal Care” created by the National Society for Medical Research and the “Guide for the Care and Use of Laboratory Animals” prepared by the National Academy of Sciences and published by the National Institutes of Health (NIH). Animal experiments were approved by the Austrian Ministry of Education, Science and Culture Division (GZ 66.011/0135-II/10b/2009) and were carried out in accordance with the approved guidelines.

### 2.2. Study Design and Animal Groups

A summary of the cohorts and study conditions are presented in Table 1. Each group included five animals. Animals were anesthetized with a combination of xylazine (Xylasol, 5 mg/kg) and ketamine (Ketavet, 100 mg/kg), injected intramuscularly. Limb transplantations including skin, muscle, bone, and vessels were performed as per a standardized technique between Brown Norway and Lewis rats (LEW) [12]. In summary, the BN donor femoral vessels were exposed and transected at the level of the inguinal ligament. The donor limb was flushed through the femoral artery with cold saline. In the next step, the limb was amputated at the level of the distal femur. The LEW recipient hind limb was prepared similarly. After bone fixation, repair of the ventral and dorsal muscle groups was performed. After anastomosis of the femoral artery and vein using 10-0 interrupted nylon suture the skin was closed with 4-0 Vicryl suture. Syngeneic limb transplantations (isografts) were used as controls. Skin biopsies from the rejecting animals as well as from the isografts were taken either from the thigh or from the footpad. Inflammatory skin reactions and control conditions were induced on the pinna as well as on the footpad of male LEW rats.

### 2.3. CHS Model Induced on the Pinna

Contact hypersensitivity was induced via epicutaneous application of the hapten 2, 4-dinitro-1-fluorobenzene (DNFB, Sigma). Rats were anesthetized using a combination of ketamine (100 mg/mL) and xylazine (20 mg/mL) prior to sensitization. LEW rats were sensitized with 100 *μ*L DNFB (1% (w/v) in 4 : 1 acetone : olive oil) applied to the skin of the shaved abdomen. On day 5, the dorsal surface of the right pinna was challenged with 50 *μ*L of DNFB (1% (w/v) in 4 : 1 acetone : olive oil) whereas the left pinna was treated with vehicle (acetone/olive oil) only. Ear thickness was monitored using a digital caliper (Kroeplin, Germany) before challenge and every day after challenge. Inflammatory response was assessed as positive reaction with double size ear swelling. Animals from POD (postoperative day) 1 to POD 5 after challenging were sacrificed and the complete pinnae were harvested and processed for further analysis.

### 2.4. DTH Model Induced on the Footpad (Planta Pedis)

The DTH reaction was induced via subcutaneous injections of mBSA (Sigma-Aldrich, Germany) using eight- to ten-week-old (200–250 g) male Lewis rats (LEW) (Charles River Laboratories, Germany). Rats were sensitized by three subcutaneous injections on the shaved abdominal skin using 300 *μ*L of 1 mg/mL mBSA in saline : CFA (=complete Freund's adjuvant) (l : l). After eight days, the stimulation was performed by injecting 100 *μ*L mBSA (10 mg/mL) subcutaneously into the right hind footpad at 2 different sites. A saline injection into the left footpad functioned as control. The swelling in both hind paws was determined 24 h later using a digital caliper. The DTH response was expressed as a percent increase of the injected paw. Skin samples from the footpad were taken at 24 h and 48 h after injection and processed for further analysis.

### 2.5. Biopsies and Sampling

Biopsies were taken under anesthesia with Xylasol and Ketavet (5 mg/kg and 100 mg/kg i.m). Skin biopsies were taken on postoperative day (POD) 5 after transplantation from the thigh as well as from the footpad (planta pedis) of five allografts and isografts. Biopsies were divided in 4 pieces and stored in RNA-later, 4% paraformaldehyde or snap-frozen in liquid nitrogen for proteomics, genomics, and histological analysis.

In the CHS and DTH groups, skin biopsies either from the pinna or the footpad were taken 24 and 48 hours after injection. All samples were processed as described above. H&E stainings showed 24 hours after injection the most impressive immunological reaction. The amount and distribution of immune cells in the CHS and DTH group at this specific time point were best comparable to grade II rejection in the allografts on POD 5.

### 2.6. Clinical and Histopathological Assessment

Animals were monitored daily for signs of rejection/inflammation by inspection. The primary clinical diagnosis of rejection was based on the following criteria. Skin rejection was classified according to a 5-graded scale, grade 0—no signs of rejection; grade I—erythema; grade II—erythema and edema; grade III—epidermolysis; grade IV—necrosis, and analyzed from POD 1 to POD 8 in the allograft group (*n* = 5). H&E-stained paraffin sections from skin biopsies were evaluated for lymphocytic infiltration according to the BANFF 2007 guidelines by a pathologist in a blinded fashion.

### 2.7. Sample Preparation, RNA Isolation, and qPCR

Samples were stored in RNA-later stabilization solution (Sigma-Aldrich, Germany) at −80°C as described above. Tissue was homogenized using TissueRuptor (Qiagen, Germany) according to the manufacturer's instructions. Total RNA was isolated from tissue samples using the RNAeasy mini kit (Qiagen, Germany) including DNAse treatment according to the supplier's instructions. Yield and purity of RNA were assessed using Nanodrop 2000c spectrophotometer (Thermoscientific, USA). RNA purity was estimated on the basis of the OD 260/280 ratio. Briefly, 1 *μ*g total RNA was reversely transcribed using the QuantiTect Reverse Transcription Kit (Qiagen, Hilden, Germany) according to the manufacturer's instructions. Quantitative real-time PCR was performed on a 7500 Real-Time PCR System (Applied Biosystems) in samples from individual animals using SYBR green (Qiagen, Germany) and in compliance with the MIQE guidelines. Reactions were set up in a total volume of 15 *μ*L per reaction containing 50 ng cDNA template, 1.5 *μ*L QuantiTect Primer Assay (Quiagen, Germany), 7,5 *μ*L QuantiTect SYBR green PCR kit (Quiagen, Germany), and 5 *μ*L ddH2O. Validated primers (QuantiTect Primer Assay) for Ifng, Il1a, Il1b, Il2, Il4, Il5, Il6, Il10, Il12a, Il12b, Il17a, Il18, chemokine ligand 2 (Ccl2), monocyte chemotactic protein 3 (Ccl7), Cxcl1, Tnf, and granulocyte-macrophage colony stimulating factor (Csf2) were obtained from Qiagen, Germany. Cycling conditions included a hot start activation (5 min, 95°C), 40 cycles of 10 sec denaturation (95°C), and annealing and extension (30 sec, 60°C). Amplicons were quantified with the comparative threshold cycle (C_T_) method, and data acquisition was performed using the 7500 System SDS Software Version 2.0.5 (Applied Biosystems). Amplification specificity was checked using melting curve according to the manufacturer's instructions. RNA quantification was carried out using the ΔC_T_ method. C_T_ values were normalized to* beta actin* as reference gene.

### 2.8. Statistics

Descriptive statistics were generated to assess quality of data. Relative expression levels (ΔC_T_) between different groups were analyzed by Mann-Whitney *U* test and presented for INF and REJ as mean + SEM (normalized to the mean in the INF group). All *P* values were adjusted for multiple hypothesis testing according to the Benjamini-Hochberg method based on the false discovery rate (FDR). Gene expression which contributes most to the separation between the two rejection and inflammatory models was determined by a linear discriminant analysis (LDA) model including all seventeen genes. All possible LDA models including only two genes at a time were evaluated based on Wilks Λ statistics. Relative expression levels of the seventeen genes (ΔC_T_) were normalized to the mean within the respective controls and isograft samples (ΔΔC_T_≈ log2-fold changes). The mean log2-fold changes within each group were visualized as heat map and hierarchical clustering was performed using Genesis. Two-way analysis of variance (ANOVA) was performed with skin type as one factor and rejection/inflammation model as the other factor, including interaction between these factors. For selected candidates differences between the individual groups were tested by a Tukey HSD post hoc analysis. All statistical analyses were performed using IBM SPSS statistics software (version 20) and the statistical software environment R.

## 3. Results

### 3.1. Clinical and Histological Evaluation of Skin Samples

**Figure 1:**
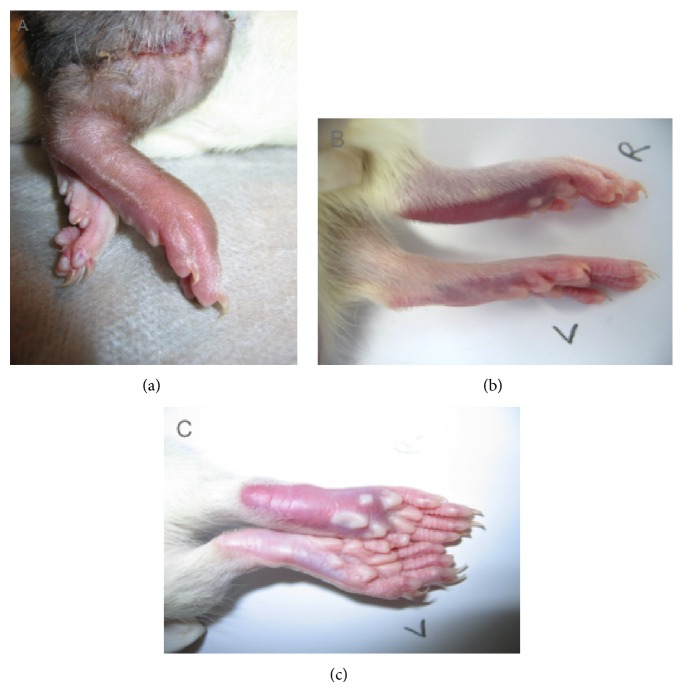
Clinical evaluation of animals. Representative pictures of skin inflammation: (a) transplanted limb showing clinically grade 2 rejection characterized by erythema and swelling; (b, c) DTH reaction on the planta pedis on the right paw and the control footpad on the left side without any inflammatory reaction.

**Figure 2:**
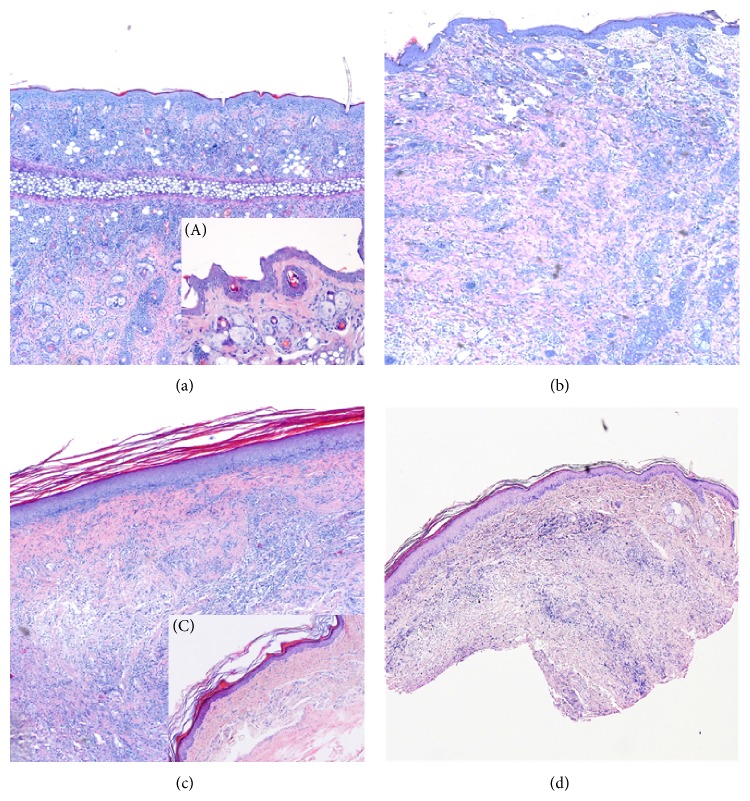
Histological evaluation of skin samples. Representative microscopic images of hematoxylin and eosin stained histological skin sections: (a) contact hypersensitivity (CHS) reaction (pinna, 24 h), (A) control skin from left pinna, (b) skin rejection (grade 2, thigh, POD 5); (c) delayed type hypersensitivity (*DTH*) reaction (planta pedis, 24 h), (C) control skin from left footpad, and (d) skin rejection (grades 1-2, planta pedis) are presented.

On POD 5, 40% of the animals displayed grade I rejection and 60% grade II rejection. The CHS and DTH animals showed a most impressive erythema and swelling 24 hours after challenge (Figure 1). Skin biopsies taken from thigh and footpad from allografts at POD 5 exhibited an almost identical composition and distribution of inflammatory cells when compared with CHS and DTH at 24 hours after challenge. Histological evaluation showed a moderate to severe perivascular inflammation with or without mild epidermal and/or adnexal epidermal dyskeratosis or apoptosis consistent with rejection grade II in 80% of the biopsies taken from thigh and footpad in the rejection group (ATC). 24 hours after challenge with DTH, animals presented with a moderate to severe inflammatory response mainly located in the epidermis as well as an interphase reaction, correlating with grade II rejection. The same histological finding was found in the CHS group, and 50% of the animals, however, showed progression to a severe inflammatory response, correlating with grade III rejection (Figure 2).

Histology of skin rejection at selected time points revealed that immune cells were distributed in a diffuse pattern in the dermis and at the dermal-epidermal interface on the thigh, whereas on the footpad the infiltrate was mainly located perivascularly in the dermis.

Skin biopsies from isografts (thigh and footpad) showed in 80% no inflammatory response on POD 5. One animal displayed a mild inflammatory reaction due to an infection. None of control animals showed any signs of skin inflammation.

### 3.2. Cytokine Expression in Acute Skin Rejection (Thigh and Footpad) versus Skin Inflammation (CHS and DTH)

**Figure 3:**
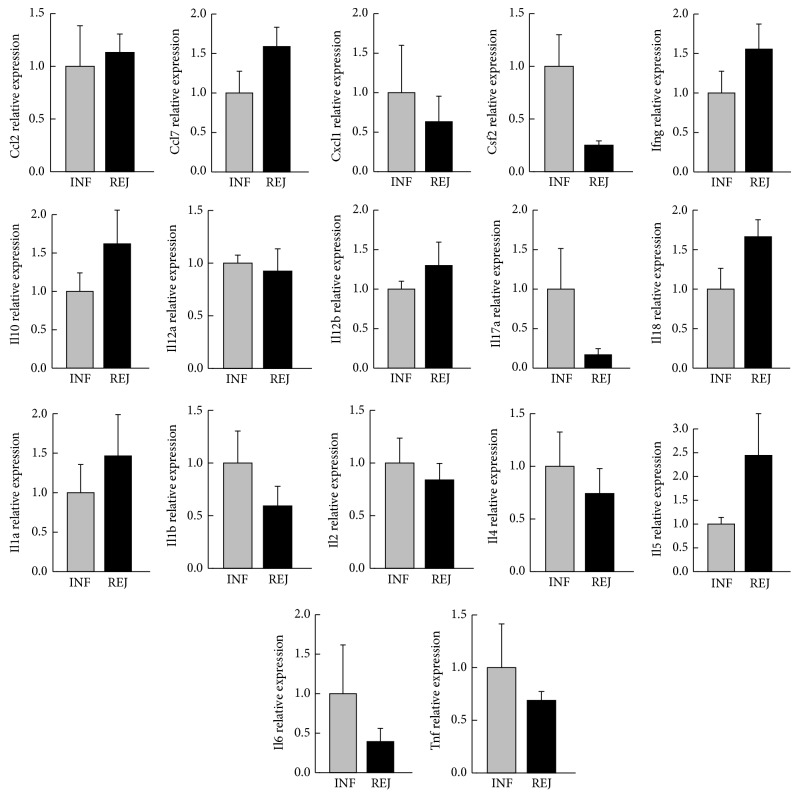
Relative expression of 17 cytokines/chemokines mRNA extracted from samples of an inflammatory disease model (DTH, CHS) or from planta pedis or thigh in a rat hind limb transplantation model on POD 5. Relative expression levels are presented as mean + standard error of mean and normalized to the mean level in the inflammatory model.

Hypothesizing that there is a distinct pattern of cytokine expression for both skin rejection and skin inflammation, we analyzed a panel of 17 inflammatory cytokines by quantitative real-time PCR in both conditions. Inflammatory skin disease models [INF: contact hypersensitivity (CHS) and delayed type hypersensitivity (DTH)] were compared to acute skin rejection in thigh and footpad (REJ). As shown in Figure 3, only few genes showed a differing relative expression between the two groups. Although* Ccl7*,* Csf2*,* Il18*, and* Il1b* were significantly differently expressed based on a rank based statistics (*P* < 0.05) they were not considered as significantly differently expressed after adjustment for multiple hypothesis testing at a FDR <5%.

**Figure 4:**
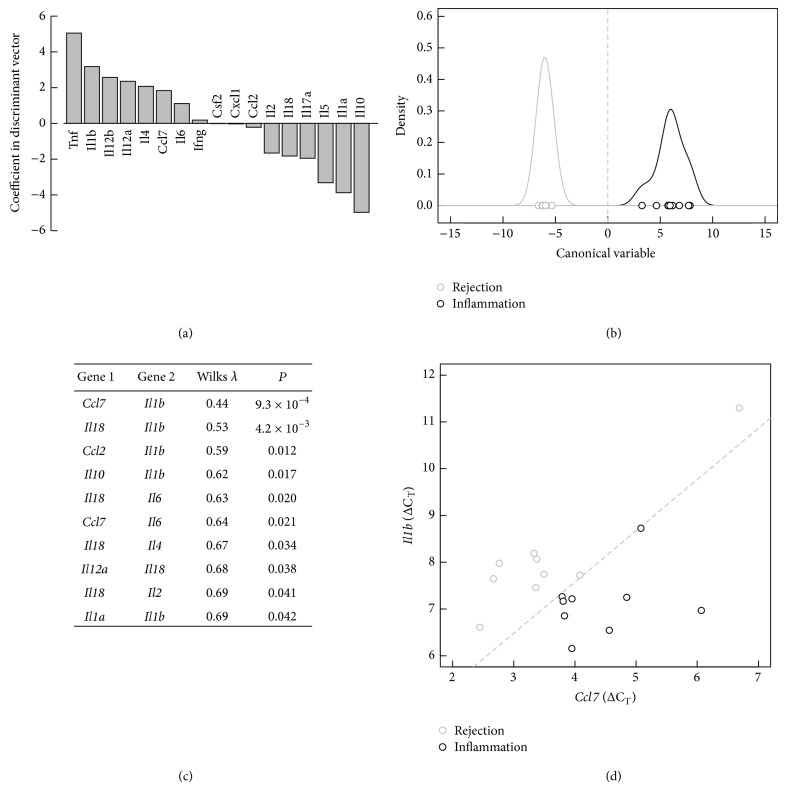
Separation of the samples within the two combined studied groups by linear discrimination analysis (LDA). (a) Coefficients of the discriminant vector from a multivariate model indicating the contribution to the separation for each of the 17 studied genes, (b) separation of the two groups by values of the canonical variable for each sample resulting from LDA including all 17 cytokines/chemokines relative mRNA levels (ΔC_T_) and corresponding density distribution, (c) list of significant LDA models when studying all pairwise gene models, and (d) linear separation based on the ΔC_T_ levels of Ccl7 and Il1b are shown. Allografts skin samples are shown in gray, inflammatory skin disease is shown in black, and dashed gray line indicates linear separation and partition.

Next, we addressed the question of whether the relative expression (ΔC_T_) of the cytokines/chemokines can be used in a multivariate model to separate the inflammatory disease models (INF) from rejection (REJ). For this purpose LDA including all chemokines/cytokines were performed and a clear separation of the two groups could be revealed (Figure 4(b)). Tnf and Il1b were the genes with the highest positive coefficients in the discriminant vector indicating a strong contribution of these two genes in the multivariate separation model (Figure 4(a)). When studying all pairwise gene models, Il1b and Ccl7 were included in the most significant LDA model (Wilks *λ* = 0.44, *P* < 0.001) and Il18 and Il1b in the second most significant LDA model (Figure 4(c)). As shown in Figure 4(d), INF and the REJ displayed a very different profile based on quantitative real-time PCR measured expression levels of Il1b and Ccl7. In summary, gene expression profiles of Ccl7, Il1b, Il18, and Tnf in the skin are highly indicative of separation of rejection from inflammatory diseases and thus are valuable for diagnosis.

### 3.3. Gene Expression Levels Differ between Skin Types and Affect the Separation between Rejection and Inflammation

**Figure 5:**
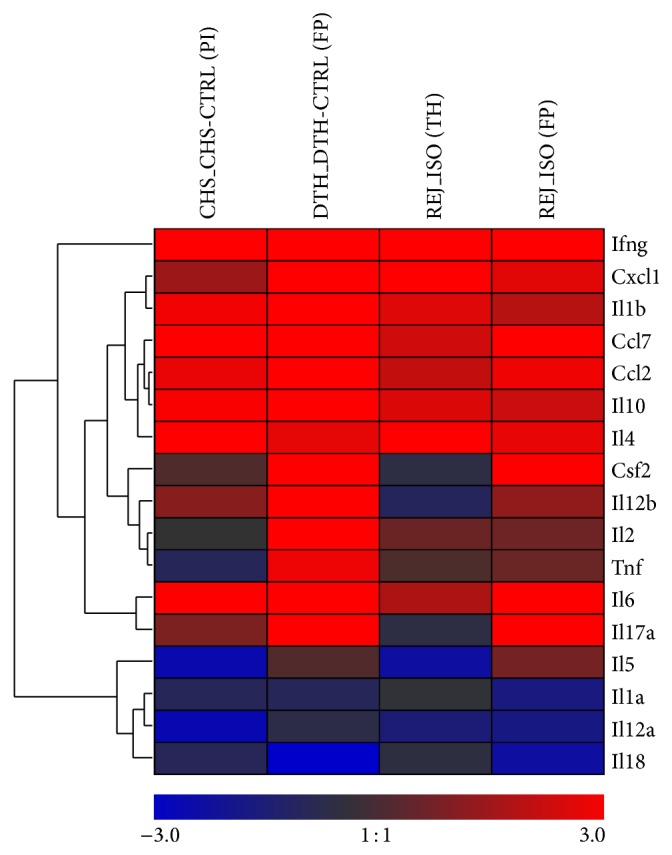
Heat map visualizing mean log2-fold changes of cytokine/chemokine mRNA levels in two different inflammatory models (DTH, CHS) and two different skin types of allograft rejection at POD 5 in rat hind limb transplantation. Relative expressions are normalized to the mean of the controls from the respective group. Greater levels of inflammatory mediators (log2-fold changes > 0) are indicated in red and smaller levels (log2-fold changes < 0) are indicated in blue according to the legend. Similar profiles of mediators across all conditions are grouped by average linkage hierarchical clustering as indicated by the dendrogram (tree) at the left.

**Figure 6:**
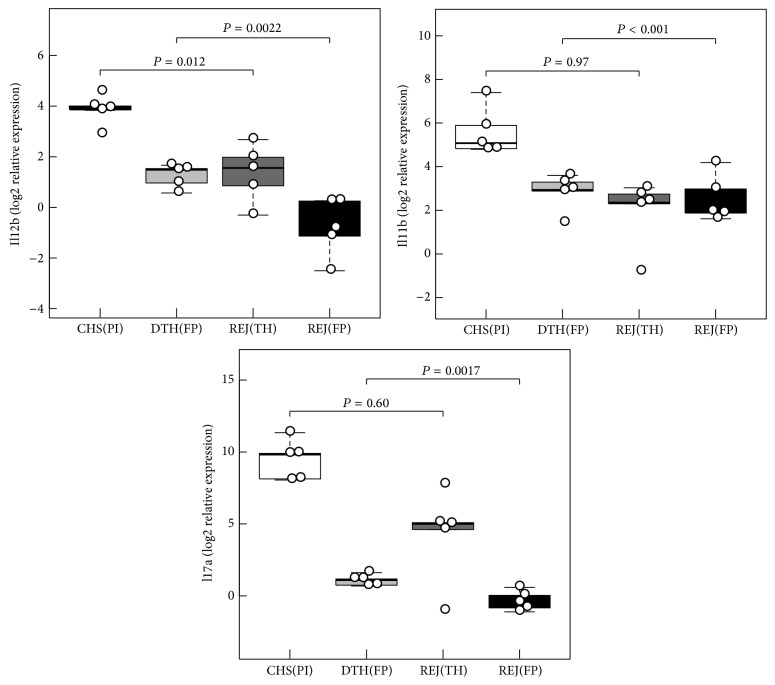
Distribution of log2-fold gene expression changes for 3 selected mediators (Il12b, Il1b, and Il17a) in the two inflammatory models (CHS, DTH) as well as in footpad (FP) and thigh (TH) samples from allograft rejection in rat hind limb transplantation. Relative expression levels are normalized to the mean of the controls in the respective group. Differences of the relative expressions (log2-fold changes) were tested between the rejection and the inflammatory model in the same skin type (hairy or nonhairy skin) by Tukey HSD post hoc procedure and resulting adjusted *P* values are provided.

Furthermore we aimed to clarify whether there are differences in cytokine gene expression patterns between skin types and if they have an impact on the distinction between rejection and inflammation. To address these questions, samples from both inflammatory models were analyzed separately, the DTH model on the pinna (hairy skin type) and the CHS model on the planta pedis (nonhairy skin type), and compared with hairy thigh skin and the nonhairy footpad skin from allografts. The relative expression levels of the 17 cytokines/chemokines (ΔC_T_) were then normalized to the mean within the respective controls and isografts (ΔΔC_T_≈ log2-fold changes). In Figure 5, a heat map is showing the mean of all log2-fold changes within each of the four groups for all genes studied. A distinct expression pattern differentiating the two anatomical sites in rejection (REJ) as well as inflammation (INF) was observed suggesting differential relative cytokine gene expression in the examined skin types. The results of this analysis indicate that for some genes the magnitude of the differences in relative expression between the skin types exceeds those between rejection and inflammation. To account for the anatomical location in the separation analysis we performed a two-way ANOVA analysis with skin type as one variable and rejection/inflammation model as the other variable and considered their interdependence. A high mutual dependence between these variables was observed for Il12a, Il2, and Tnf (interaction *P* < 0.001) (Table 2). Since these cytokines were also highly significantly differentially expressed between the two skin types this indicates that the differences between rejection and inflammation are systematically biased by the anatomical location. Il12b, Il17a, and Il1b gene expression levels significantly differ between the rejection and inflammatory disease models with only moderate interaction and were independent from skin types (Figure 6 and Table 2). To test for differences in the profile of these three genes between the individual groups a Tukey HSD post hoc procedure was performed. As shown in Figure 6 the expression levels of these three genes differ significantly on the footpad between DTH inflammation model and the rejection group (Il12b,* adjusted P* = 0,0022; Il1b,* adjusted P* = 7.8 × 10^−4^; Il17a,* adjusted P* = 0,0017). Moreover, Il12b distinguished contact hypersensitivity from rejection in the thigh skin (*adjusted P* = 0,012).

## 4. Discussion

Episodes of acute skin rejection are a common and serious complication after VCA. Since the macroscopic pattern of skin rejection is rather heterogeneous, differential diagnosis towards T-cell mediated inflammatory skin diseases can be difficult [2]. Moreover, atypical forms of skin rejection have been recorded, which mainly manifested on the palmar hand and the radial side of the fingers (nonhairy skin) [13]. Based on these clinical observations, we hypothesized that localization specific immune mechanisms orchestrate leukocyte migration and infiltration in different skin types during skin rejection as well as during T-cell mediated skin inflammation. We also postulated that acute skin rejection can be diagnosed by analyzing expression patterns of inflammatory mediators. Applying a rat hind limb allotransplant model as well as two rodent models for inflammatory skin diseases, we demonstrated statistically significant differences in gene expression patterns between our study groups. Furthermore, gene expression levels differ between different skin types and affect the separation of the experimental groups. Previously assessed protein expression profile does not correlate well with the gene expression analysis presented here. Our initial data on protein values identified IL-12p70 and TNF-*α* as major discriminators between skin rejection and inflammatory skin reactions on POD 5. Principal component analysis (PCA) on protein data identified IL-1*α*, GM-CSF, IL-6, and IL-18 as key drivers of skin rejection in the same study setting (paper submitted). Interestingly, when we performed PCA on the combined mean-centered protein dataset including allografts, isografts, and immunosuppressed animals, along 4 different time-points (POD 3–9) in skin and muscle, IL-1*α*, IL-18, IL-1*β*, and IL-4 were identified as principal drivers of transplant rejection [11]. On RNA level, Tnf was also identified as important marker in the discriminant vector indicating a strong contribution in the multivariate separation model; however, when studying all pairwise gene models, measurements of Ccl7, Il1b, and Il18 were most significant for differentiation between skin rejection and skin inflammation on POD 5 (Figure 4) on mRNA level. Considering diverse skin locations, Il12b best distinguished the contact hypersensitivity group from rejection.

A discrepancy between mRNA and protein levels with overall correlation of *r* ≤ 0.4 is evident from the literature. Similar observations have been made across several cell lines, cell types, organisms, and even more complex* in vivo* situations like human cancer tissues [14]. This might apply specifically to regulatory proteins including transcription factors and secreted proteins but not to housekeeping genes [15]. There are several reasons for the limited correlation between mRNA and protein levels including different* in vivo* half-lives of proteins and mRNAs and various mechanisms for posttranscriptional modification [16].

Moreover, the partial concordance of the drawn conclusions might demonstrate that the identification of marker proteins or genes for skin rejection is strongly associated with specific time frames of biopsy retrieval.

In synopsis of the RNA and protein data, the Il1 family holds great potential to differentiate skin rejection from the inflammatory skin diseases used as models in this experimental study design. Furthermore, targeting the Il1 family appears a promising option for a new therapeutic regimen to treat skin rejection episodes in reconstructive transplantation. A first step would be to use anakinra, a specific inhibitor of both IL-1*α* and IL-1*β* already clinically applied for the treatment of rheumatoid arthritis in humans [17]. In the present study we identified differences in cytokine expression between hair baring and nonhairy skin. The analyses showed a distinct expression pattern, clearly differentiating the two anatomical sites in rejection as well as in the inflammatory skin disease models suggesting differential relative cytokine gene expression in the examined skin types and situations. For some genes, however, the differences in relative expression levels between the skin types might be even higher than those between rejection and inflammation. In a subsequent trial, cytokine protein expression between three defined skin sampling localizations (thigh, dorsum, and planta pedis) in a rat limb allograft was assessed. In a pairwise study design eight out of 14 analytes were significantly differentially expressed in hairy (thigh, dorsum) versus nonhairy (planta pedis) skin, with MCP-1, IL-4, and GRO-KC exhibiting the greatest individual effects between both skin types. This observation further strengthens the theory of a skin type specific inflammatory milieu during skin rejection and provides a possible explanation for the observed atypical rejection patterns in hand transplant patients. A further explanation for this phenomenon is the specialized properties and functions of the nonhairy skin. The palmar and plantar skin are relatively thick with a prominent stratum corneum. The position on feet and hand results in exposure to mechanical stress and chemical irritation. Hence, it might be speculated that this type of skin innately is less vulnerable to inflammation and rejection [18].

## 5. Conclusion

Although our study remains observational, we herein aimed to provide information, which could help to identify new diagnostic markers and novel targets for the treatment of acute skin rejection. Early and specific diagnosis as well as targeted therapy of skin rejection, namely, selectively blocking leukocyte recruitment during rejection, could evolve to a promising option for VCA patients, lowering their burden of long-term immunosuppression.

## Figures and Tables

**Table 1:** Study groups and design.

Group and number of animals	Surgery procedure	Biopsy time point (day after transplantation)	Skin type	Biopsy region	Sample types provided
1 (ATC) *n* = 5 (thigh) *n* = 5 (footpad)	Orthotopic hind limb transplantation	Postoperative day (POD) 5	Hairy skin	Thigh	Allografts without immunosuppression
			Nonhairy skin	Footpad	Allografts without immunosuppression

2 (ISO) *n* = 5 (thigh) *n* = 5 (footpad)	Orthotopic hind limb transplantation	Postoperative day (POD) 5	Hairy skin	Thigh	Isografts
			Nonhairy skin	Footpad	Isografts

3 (CHS) *n* = 5	CHS reaction on ear	24 hours after challenge	Hairy skin	Pinna	Skin inflammation

4 (CHS-CTRL) *n* = 5	CHS reaction on ear	24 hours after challenge	Hairy skin	Pinna	Skin inflammation (control)

5 (DTH) *n* = 5	DTH reaction on footpad	24 hours after challenge	Nonhairy skin	Footpad	Skin inflammation

6 (DTH-CTRL) *n* = 5	DTH reaction on footpad	24 hours after challenge	Nonhairy skin	Footpad	Skin inflammation (control)

ATC: rejection group (donor: brown Norway (BN) rat, recipient: Lewis (LEW) rat, no immunosuppression).

ISO: isograft group (donor: LEW rat, recipient: LEW rat, syngeneic control group).

CHS: contact hypersensitivity group.

CHS-CTRL: contact hypersensitivity control group.

DTH: delayed type hypersensitivity group.

DTH-CTRL: delayed type hypersensitivity control group.

**Table 2:** Results from two-way analysis of variances (ANOVA) of log2-fold changes of gene expression considering the skin type (hairy or nonhairy skin) and the model (allograft rejection or inflammatory skin disease) as factors and their potential interaction.

Gene	Factor	Df	Sum Sq	Mean Sq	*F* value	*P*	Sign.
*Il12b *	SKIN_TYPE	1	28.20	28.20	35.38	2.0 × 10^−5^	∗∗∗
	MODEL	1	25.60	25.60	32.13	3.5 × 10^−5^	∗∗∗
	SKIN_TYPE:MODEL	1	0.26	0.26	0.33	0.57	

*Il17a *	SKIN_TYPE	1	211.69	211.69	65.61	4.7 × 10^−7^	∗∗∗
	MODEL	1	54.56	54.56	16.91	8.2 × 10^−5^	∗∗∗
	SKIN_TYPE:MODEL	1	17.54	17.54	5.44	0.033	∗

*Il1b *	SKIN_TYPE	1	5.95	5.95	4.32	0.054	.
	MODEL	1	19.71	19.71	14.31	0.0016	∗∗
	SKIN_TYPE:MODEL	1	14.00	14.00	10.17	0.0057	∗∗

*Il12a *	SKIN_TYPE:MODEL	1	6.76	6.76	18.63	5.3 × 10^−4^	∗∗∗

*Il2 *	SKIN_TYPE:MODEL	1	16.18	16.18	17.04	7.9 × 10^−4^	∗∗∗

*Tnf *	SKIN_TYPE:MODEL	1	11.82	11.82	22.81	2.1 × 10^−4^	∗∗∗

Sign: significance score: (^***^ *P* < 0.001, ^**^ *P* < 0.01, ^*^ *P* < 0.05, and ^.^ *P* < 0.1), Df: degree of freedom.

Sum Sq: sum of squares, Mean Sq: mean of sum of squares.

Log2-fold changes were related to the mean of the controls in the respective groups. Results are shown for genes with most significant effect of the factor model (rejection versus inflammation) and only moderate interaction with the factor skin type, as well as for genes with the most significant statistical interaction between the two factors.

## References

[1] Cendales L. C., Kanitakis J., Schneeberger S. (2008). The Banff 2007 working classification of skin-containing composite tissue allograft pathology. *The American Journal of Transplantation*.

[2] Kanitakis J. (2008). The challenge of dermatopathological diagnosis of composite tissue allograft rejection: a review. *Journal of Cutaneous Pathology*.

[3] Saint-Mezard P., Berard F., Dubois B., Kaiserlian D., Nicolas J. F. (2004). The role of CD4+ and CD8+ T cells in contact hypersensitivity and allergic contact dermatitis. *European Journal of Dermatology*.

[4] Pastore S., Mascia F., Mariotti F., Dattilo C., Girolomoni G. (2004). Chemokine networks in inflammatory skin diseases. *European Journal of Dermatology*.

[5] Grabbe S., Schwarz T. (1998). Immunoregulatory mechanisms involved in elicitation of allergic contact hypersensitivity. *Immunology Today*.

[6] Black C. A. (1999). Delayed type hypersensitivity: current theories with an historic perspective. *Dermatology Online Journal*.

[7] Hautz T., Zelger B., Grahammer J. (2010). Molecular markers and targeted therapy of skin rejection in composite tissue allotransplantation. *The American Journal of Transplantation*.

[8] Kanitakis J., Jullien D., Petruzzo P. (2003). Clinicopathologic features of graft rejection of the first human hand allograft. *Transplantation*.

[9] Watanabe H., Unger M., Tuvel B., Wang B., Sauder D. N. (2002). Contact hypersensitivity: the mechanism of immune responses and T cell balance. *Journal of Interferon and Cytokine Research*.

[10] Wood K. J., Goto R. (2012). Mechanisms of rejection: current perspectives. *Transplantation*.

[11] Wolfram D., Starzl R., Hackl H. (2014). Insights from computational modeling in inflammation and acute rejection in limb transplantation. *PLoS ONE*.

[12] Sacks J. M., Kuo Y.-R., Horibe E. K. (2012). An optimized dual-surgeon simultaneous orthotopic hind-limb allotransplantation model in rats. *Journal of Reconstructive Microsurgery*.

[13] Schneeberger S., Gorantla V. S., van Riet R. P. (2008). Atypical acute rejection after hand transplantation. *American Journal of Transplantation*.

[14] Lichtinghagen R., Musholt P. B., Lein M. (2002). Different mRNA and protein expression of matrix metalloproteinases 2 and 9 and tissue inhibitor of metalloproteinases 1 in benign and malignant prostate tissue. *European Urology*.

[15] Schwanhüusser B., Busse D., Li N. (2011). Global quantification of mammalian gene expression control. *Nature*.

[16] Greenbaum D., Colangelo C., Williams K., Gerstein M. (2003). Comparing protein abundance and mRNA expression levels on a genomic scale. *Genome Biology*.

[17] Mertens M., Singh J. A. (2009). Anakinra for rheumatoid arthritis: a systematic review. *Journal of Rheumatology*.

[18] Hautz T., Wolfram D., Eberhart N. (2014). The impact of skin type and area on skin rejection in limb transplantation. *Vascularized Composite Allotransplantation*.

